# Residual risk prediction in anticoagulated patients with atrial fibrillation using machine learning: A report from the GLORIA‐AF registry phase II/III

**DOI:** 10.1111/eci.14371

**Published:** 2024-12-11

**Authors:** Yang Liu, Yang Chen, Ivan Olier, Sandra Ortega‐Martorell, Bi Huang, Hironori Ishiguchi, Ho Man Lam, Kui Hong, Menno V. Huisman, Gregory Y. H. Lip, the GLORIA‐AF Investigators

**Affiliations:** ^1^ Liverpool Centre for Cardiovascular Science at University of Liverpool, Liverpool John Moores University, and Liverpool Heart & Chest Hospital Liverpool UK; ^2^ Department of Cardiovascular Medicine, the Second Affiliated Hospital, Jiangxi Medical College Nanchang University Nanchang Jiangxi China; ^3^ Data Science Research Centre Liverpool John Moores University Liverpool UK; ^4^ Department of Cardiology The First Affiliated Hospital of Chongqing Medical University Chongqing China; ^5^ Division of Cardiology, Department of Medicine and Clinical Science Yamaguchi University Graduate School of Medicine Ube Japan; ^6^ Department of Genetic Medicine the Second Affiliated Hospital of Nanchang University Nanchang Jiangxi China; ^7^ Jiangxi Key Laboratory of Molecular Medicine the Second Affiliated Hospital of Nanchang University Nanchang China; ^8^ Department of Medicine – Thrombosis and Hemostasis Leiden University Medical Center Leiden The Netherlands; ^9^ Department of Clinical Medicine Aalborg University Aalborg Denmark

**Keywords:** atrial fibrillation, machine learning, oral anticoagulant, residual risk

## Abstract

**Background:**

Although oral anticoagulation decreases the risk of thromboembolism in patients with atrial fibrillation (AF), a residual risk of thrombotic events still exists. This study aimed to construct machine learning (ML) models to predict the residual risk in these patients.

**Methods:**

Patients with newly diagnosed non‐valvular AF were collected from the Global Registry on Long‐Term Oral Anti‐Thrombotic Treatment in Patients with Atrial Fibrillation (GLORIA‐AF) registry. To predict the residual risk of the composite outcome of thrombotic events (defined as ischemic stroke, systemic embolism, transient ischemic attack and myocardial infarction), we constructed four prediction models using the logistic regression (LR), random forest, light gradient boosting machine and extreme gradient boosting machine ML algorithms. Performance was mainly evaluated by area under the receiver‐operating characteristic curve (AUC), g‐means and F1 scores. Feature importance was evaluated by SHapley Additive exPlanations.

**Results:**

15,829 AF patients (70.33 ± 9.94 years old, 55% male) taking oral anticoagulation were included in our study, and 641 (4.0%) had residual risk, sustaining thrombotic events. In the test set, LR had the best performance with higher AUC trend of 0.712. RF has highest g‐means of 0.295 and F1 score of 0.249. This was superior when compared with the CHA_2_DS_2_‐VA score (AUC 0.698) and 2MACE score (AUC 0.696). Age, history of TE or MI, OAC discontinuation, eGFR and sex were identified as the top five factors associated with residual risk.

**Conclusion:**

ML algorithms can improve the prediction of residual risk of anticoagulated AF patients compared to clinical risk factor‐based scores.

## Graphical abstract

This study aimed to construct machine learning (ML) models in patients with atrial fibrillation (AF) to predict the residual risk from the Global Registry on Long‐Term Oral Anti‐Thrombotic Treatment in Patients with Atrial Fibrillation (GLORIA‐AF) registry. We found ML algorithms can improve the prediction of residual risk of anticoagulated AF patients compared to clinical risk factor‐based scores (CHA_2_DS_2_‐VA and 2MACE). Age, history of TE or MI, OAC discontinuation, eGFR and sex were identified as the top 5 predictors associated with residual risk.

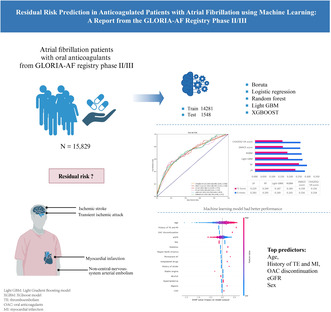

## 1 INTRODUCTION

Atrial fibrillation (AF) is the most common cardiac arrhythmia globally and is associated with increased morbidity and mortality from thrombotic events, including ischemic stroke, systemic embolism, transient ischemic attack, and myocardial infarction.^1^ Thus, AF poses a significant burden on healthcare systems worldwide.

Oral anticoagulant (OAC) therapy has a well‐established role in the management of AF to reduce the risk of these thromboembolic events.^2^ Despite the effectiveness of anticoagulation, a considerable proportion of AF patients continue to experience adverse outcomes, including stroke and systemic embolism.^3^, ^4^, ^5^, ^6^ At approximately 2.2 years of follow‐up, the annual stroke risk remains around 1.7% for warfarin and 1.4% for non–vitamin K antagonist oral anticoagulants.^4^ Although the residual risk of stroke in OAC‐treated patients is comparatively low, its population burden remains high given the frequency of AF and the significant consequences of AF‐related stroke.^6^, ^7^ Residual risk assessment might be beneficial for the secondary prevention of these patients, but how best to predict this residual risk remains uncertain.

Machine learning (ML) has emerged as a powerful tool in healthcare analytics, capable of identifying intricate patterns and relationships within large and complex datasets, including thrombotic and bleeding events.^8^, ^9^, ^10^, ^11^ The Global Registry on Long‐Term Oral Anti‐Thrombotic Treatment in Patients with Atrial Fibrillation (GLORIA‐AF) Registry represents a comprehensive collection of clinical data from up to 56,000 newly diagnosed nonvalvular AF patients in nearly 50 countries.^12^

This study aimed to explore the utility of ML techniques in predicting residual risk in AF patients receiving OAC therapy, utilizing data from the GLORIA‐AF Registry Phase II/III. By harnessing the predictive capabilities of ML, we seek to improve risk stratification, inform clinical decision‐making, and ultimately enhance outcomes for AF patients undergoing OAC therapy.^13^

## 2 METHODS

### 2.1 Data resources

This study collected AF patients who received OAC from the GLORIA‐AF Phase II and III registry. GLORIA‐AF is a prospective, multicentre global registry involving patients with newly diagnosed non‐valvular AF (<3 months before baseline visit). The study design has been previously reported.^12^, ^14^ The patients enrolled in phase II who initiated dabigatran have a 2‐year follow‐up, whereas all patients who received antithrombotic treatment enrolled in phase III had a 3‐year follow‐up period. During the follow‐up period, data on major events, concomitant diseases, cardiovascular interventions and periprocedural anticoagulation regimens were collected during scheduled hospital visits by the investigator in the Appendix S1.

### 2.2 Inclusion and exclusion criteria

In our GLORIA‐AF analysis, we included non‐valvular AF patients and excluded missing values referred to OAC therapy and outcomes on residual risk.

### 2.3 Data extraction and missing data process

At baseline, we included age, sex, race, smoking and drinking status (with current or historical status considered as positive events), type of AF (paroxysmal, persistent, permanent), clinical scores, comorbidities and history of clinical events (history of thromboembolism [TE, including stroke, systemic embolism and transient ischemic attack], myocardial infarction [MI], stroke, bleeding, as reported in the case report form by the investigator), OAC therapy and antiplatelet therapy. The variables with missing rates above 20% were excluded.

Finally, we obtained 43 variables. Missing values of continuous variables, including age, eGFR and 2MACE score, were imputed using multiple imputation by chained equations (MICE).^15^, ^16^, ^17^ eGFR was calculated by the simplified MDRD equation.^18^

### 2.4 Residual risk definitions

This study aimed to explore the risk of residual thrombotic events, defined as the composite outcome of ischemic stroke (IS), non‐central‐nervous‐system arterial embolism, transient ischemic attack (TIA) and myocardial infarction (MI). In this study, the categorization of ischemic cause was established using computed tomography or magnetic resonance scanning, or autopsy.^19^ MI was defined as the development of significant Q‐waves in at least 2 adjacent electrocardiogram leads, or at least 2 of the following three criteria: (1) typical prolonged severe chest pain of at least 30 min; (2) electrocardiographic changes suggestive of myocardial infarction including ST‐changes or T‐wave inversion in the electrocardiogram; (3) elevation of troponin or creatinine kinase‐MB to more than upper level of normal or, if creatinine kinase‐MB was elevated at baseline, re‐elevation to more than a 50% increase above the previous level, as reported in the previous study.^19^, ^20^ Stroke was defined as an acute onset of a focal neurological deficit of presumed vascular origin lasting for 24 h or more or resulting in death, including ischemic stroke, haemorrhagic stroke, and uncertain classification.

### 2.5 Feature selection

We randomly split the dataset into training and test sets by 9:1. The ratio of positive to negative events in our dataset was approximately 1:24, which is unbalanced. Automatic feature selection was performed to reduce the risk of overfitting due to the high number of variables in the original data.^21^

We utilized Boruta and LASSO for feature selection in the training set. Boruta is a wrapper algorithm employing random forests to measure feature importance, selecting those surpassing randomized variables.^22^, ^23^, ^24^, ^25^ LASSO regression uses L1 regularization to remove or reduce the impact of variables that it deems irrelevant to the outcome prediction.^26^ Features highlighted by both methods were included in the study.

Then, to avoid variable collinearity and multicollinearity, the Spearman coefficient was calculated to exclude the variables that have stronger correlations (*R* > =0.6) with others, and values with VIF >5 would be excluded. After pre‐processing and extraction features, we input 15 variables to construct models.

### 2.6 Model development

In this study, we used four algorithms to predict the occurrence of residual risk, including ML Logistic Regression (LR), Random Forest (RF), Light Gradient Boosting Machine (LGBM) and Extreme Gradient Boosting Machine (XGBM). The Grid Search method with ten‐fold cross‐validation was used to optimize the hyperparameters of ML models. In addition, we compared the performances of our ML models with CHA_2_DS_2_‐VA^27^ and 2MACE scores^28^ reported in previous studies, both based on clinical risk factors, which were calculated as previously described.^29^, ^30^

### 2.7 Model interpretation and feature importance

Receiver operating characteristic (ROC) curves were plotted, and their respective areas under the ROC curve (AUC) were calculated to evaluate the performance of ML models and compared by the DeLong test.^31^ AUC 95% confidence interval (CI) was computed using 1000 bootstrapping iterations. Further performance metrics such as accuracy, specificity, sensitivity, precision, recall, F1‐score and G‐mean were also calculated by adjusting the ROC cutoff point for each model, and their results were compared with the ones produced by the CHA_2_DS_2_‐VA score model and 2MACE score model, respectively. Considering the imbalance in our data, the G‐mean was seen as a key metric since it simultaneously considers the performance of both positive and negative classes.^32^

We then calculated the SHapley Additive exPlanations (SHAP) values, which are increasingly used for interpreting ML predictions of Light GBM and XGboost model.^33^ SHAP values encode the importance a model attributes to each feature. Utilizing this contribution information, we order the features based on their importance.^34^ SHAP offers a transparent explanation of each feature's contribution to disease diagnosis. This can significantly aid in clinical decision‐making and improve the interpretability of machine learning models.^24^

### 2.8 Univariate statistical analysis

Continuous variables were represented by mean (standard deviation [SD]) or median (interquartile range [IQR]), and categorical variables were represented by frequencies and percentages (*n* [%]). To evaluate different groups, Kruskal–Walli's test was performed for continuous variables. Categorical variables were evaluated by Pearson's chi‐squared test.

Data preprocessing, feature selection, and data cleaning were completed using R version 4.3.2 with the package ‘mice’, ‘Boruta’ and ‘lasso’ while model construction, model training and testing, as well as SHAP interpretation, were performed using Python version 3.1.2 with packages for Scikit‐learn (version 1.4.0), lightgbm (version 4.3.0), xgboost (version 2.0.3) and SHapley Additive exPlanation (version 3.11.4).

## 3 RESULTS

### 3.1 Baseline characteristics

Overall, 15,829 AF patients were included in our study (age 70.33 ± 9.94 years, 55% male). During a follow‐up of median 1176 (IQR 228–1545) days, there were 641 (4.0%) ‘residual risk’ thrombotic events in these anticoagulated patients, including 243 (1.5%) IS, 265 (1.7%) MI, 134 (0.8%) TIA and 31 (0.2%) systemic embolism (Table 1).

**TABLE 1:** Baseline characteristics of AF patients by residual risk.

Characteristic	Overall *N* = 15,829	No residual risk *N* = 15,188	Residual risk *N* = 641	*p* Value
Age, *n* (%)				
Mean (SD)	70.33 (9.94)	70.18 (9.96)	74.01 (8.83)	<0.001
Median (25%, 75%)	71.00 (65.00,78.00)	71.00 (64.00,77.00)	75.00 (69.00,81.00)	
Sex				
Male	8642 (55%)	8271 (54%)	371 (58%)	0.088
Female	7187 (45%)	6917 (46%)	270 (42%)	
BMI, kg/cm^2^				
Mean (SD)	28.87 (5.97)	28.89 (5.97)	28.47 (5.90)	0.068
Median (25%, 75%)	27.78 (24.82,31.83)	27.80 (24.84,31.84)	27.55 (24.35,31.63)	
Region NA, *n* (%)	4164 (26%)	3966 (26%)	198 (31%)	0.007
Permanent AF, *n* (%)	1659 (10%)	1572 (10%)	87 (14%)	0.009
Smoke, *n* (%)	6475 (41%)	6173 (41%)	302 (47%)	0.001
Alcohol, *n* (%)	4574 (29%)	4407 (29%)	167 (26%)	0.10
eGFR, mL/min				
Mean (SD)	77.36 (22.97)	77.58 (22.86)	72.25 (24.85)	<0.001
Median (25%, 75%)	75.85 (62.90,89.83)	76.04 (63.21,89.97)	70.28 (56.01,86.74)	
CHA_2_DS_2_‐VA score				
Mean (SD)	2.63 (1.32)	2.66 (1.34)	3.39 (1.47)	<0.001
Median (IQR)	3.00 (2.00–3.00)	3.00 (2.00–3.00)	3.00 (2.00–4.00)	
2MACE score				
Mean (SD)	1.54 (1.42)	1.51 (1.41)	2.23 (1.57)	<0.001
Median (IQR)	2.00 (0.00–2.00)	1.00 (0.00–2.00)	2.00 (1.00–3.00)	
Comorbidities and clinical end events, *n* (%)				
Hypertension	12,077 (76%)	11,561 (76%)	516 (80%)	0.011
Diabetes	3724 (24%)	3522 (23%)	202 (32%)	<0.001
Hyperlipidaemia	6755 (43%)	6423 (42%)	332 (52%)	<0.001
Coronary artery disease	2830 (18%)	2646 (17%)	184 (29%)	<0.001
Congestive heart failure	3470 (22%)	3309 (22%)	161 (25%)	0.046
Peripheral artery disease	437 (2.8%)	396 (2.6%)	41 (6.4%)	<0.001
COPD	989 (6.2%)	927 (6.1%)	62 (9.7%)	<0.001
Cancer	1565 (9.9%)	1495 (9.8%)	70 (11%)	0.4
Stable angina	959 (6.1%)	916 (6.0%)	43 (6.7%)	0.5
History of stroke	1598 (10%)	1467 (9.7%)	131 (20%)	<0.001
History of TE and MI	3256 (21%)	2999 (20%)	257 (40%)	<0.001
Therapy, *n* (%)				
Antiplatelet drugs	2855 (18%)	2685 (18%)	170 (27%)	<0.001
ACE‐I	5020 (32%)	4799 (32%)	221 (34%)	0.12
ARB	4276 (27%)	4104 (27%)	172 (27%)	>0.9
β‐blocker	10,220 (65%)	9791 (64%)	429 (67%)	0.2
Statin	7315 (46%)	6953 (46%)	362 (56%)	<0.001
Clopidogrel	634 (4.0%)	589 (3.9%)	45 (7.0%)	<0.001
Diuretic use	6218 (39%)	5941 (39%)	277 (43%)	0.037
Antiarrhythmic drugs	4001 (25%)	3871 (25%)	130 (20%)	0.003
Amiodarone	2081 (13%)	2006 (13%)	75 (12%)	0.3
Flecainide	496 (3.1%)	482 (3.2%)	14 (2.2%)	0.2
Propafenone	524 (3.3%)	513 (3.4%)	11 (1.7%)	0.021
SSRI use	632 (4.0%)	597 (3.9%)	35 (5.5%)	0.053
Digoxin	1387 (8.8%)	1323 (8.7%)	64 (10.0%)	0.3
PPI	4002 (25%)	3794 (25%)	208 (32%)	<0.001
H2 blocker	463 (2.9%)	445 (2.9%)	18 (2.8%)	0.9
Pacemaker in situ	539 (3.4%)	512 (3.4%)	27 (4.2%)	0.3
Anticoagulation	12,439 (79%)	11,944 (79%)	495 (77%)	0.4
Follow up duration, days				
Mean (SD)	1004.65 (235.44)	1006.38 (233.32)	963.63 (278.22)	0.002
Median (25%, 75%)	1091.00 (888.00,1129.00)	1092.00 (896.00,1129.00)	1086.00 (796.00,1122.00)	
Outcome events				
Composite residual risk	641 (4.0%)		641 (100%)	
Ischemic stroke	243 (1.5%)	–	243 (37.9%)	
Myocardial infarction	265 (1.7%)	–	265 (41.3%)	
Non‐central‐nervous‐system arterial embolism	31 (0.2%)	–	31 (4.8%)	
Transient ischemic attack	134 (0.8%)	–	134 (20.9%)	

*Note*: Continuous variables were presented by Mean (SD) and Median (IQR). Catalogue variables were presented by frequency and percentage (*n* %).

Abbreviations: ACE‐I, angiotensin‐converting enzyme inhibitors; ARB, angiotensin II receptor blockers; BMI, body mass index; COPD, chronic obstructive pulmonary disease; eGFR, estimated glomerular filtration rate; IQR, interquartile range; MI, myocardial infarction; PPI, Proton pump inhibitors; SD, standard deviation; SSRI, Selective Serotonin Reuptake Inhibitor; TE, thromboembolism; VKA, Vitamin K antagonists.

Patients with residual risk were older, had more females, lower eGFR. They also had a higher prevalence of permanent AF, diabetes, hyperlipidaemia, coronary artery disease, stroke, and a history of MI or TE compared to those without residual risk. Additionally, more patients with residual risk received statin, digoxin, and clopidogrel, and fewer received antiarrhythmic drugs (Table 1). There were no significant differences in baseline variables between the two groups, suggesting that the training and test sets were comparable (all *p* > 0.05, Table S1).

### 3.2 Feature selection in the training set

**FIGURE 1:**
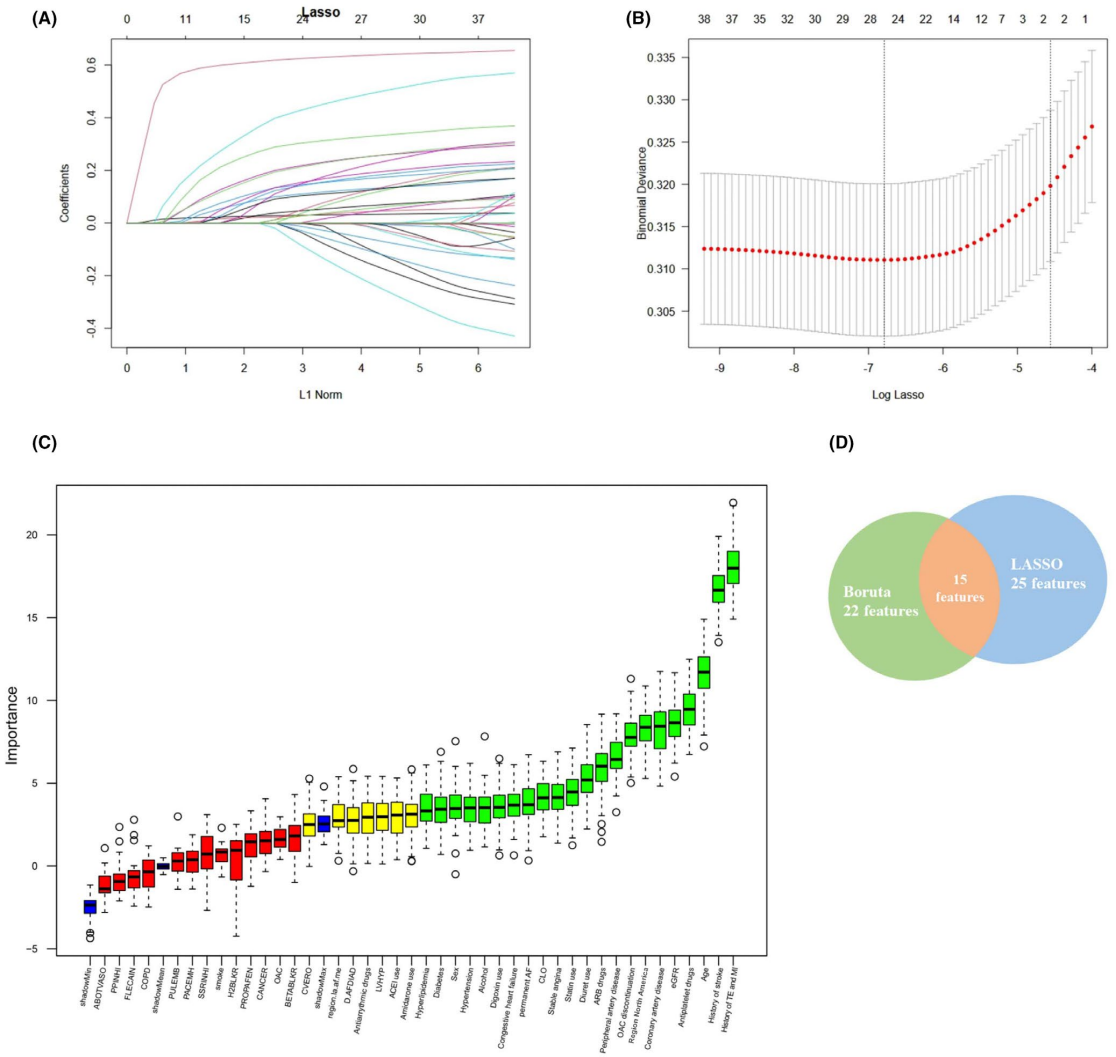
Feature selection strategy. (A) Tuning parameter selection of LASSO‐based regression model; (B) Coefficient were generated based on the optimal tuning parameter; (C) Boruta model. The horizontal axis is the name of each variable, and the vertical axis is the Z value of each variable. The box plot shows the Z value of each variable during model calculation. The green boxes represent important variables, and the yellow boxes represent possible variables, and the red boxes represent unimportant variables. (D) Common predictors between Boruta and LASSO. ABOTVASO, Aalpha blocker or other vasodilator use; ACEI, angiotensin‐converting enzyme inhibitors; ARB, Aangiotensin II receptor blockers; CHF, Ccongestive heart failure; COPD, chronic obstructive pulmonary disease; CVERO, previous cardioversion; CVHPAD, peripheral artery disease; GFR, estimated Glomerular Filtration Rate; LVHYP, Left ventricular hypertrophy; MI, myocardial infarction; NOAC, Non‐vitamin K antagonist oral anticoagulant; OAC, anticoagulants use (NOAC vs. VKA); PPI, proton pump inhibitor; SSRI, Selective Serotonin Reuptake Inhibitor; TE, thromboembolism; VKA, vitamin K antagonist.

Figure S1 shows the Spearman analysis for all variables we included, and there is no strong correlation for most variables (*R* < 0.6), except amiodarone use and antiarrhythmic drugs (*R* = 0.67). Figure 1 illustrates the strategies to find important features. The Boruta method selected 22 features while Lasso obtained 25 features. We then included 15 features in our study, which were important in both Boruta and Lasso, including 2 continuous variables (age, eGFR) and 13 categoric variables, including sex, diabetes, hyperlipidaemia, region of North America, permanent AF, alcohol, coronary artery disease, stable angina, history of stroke, history of TE or MI, OAC discontinuation, antiplatelet drugs and digoxin use. Figure S2 showed that there was no strong collinearity and multicollinearity among selected features (all *R* < 0.6, all VIF <5).

### 3.3 Model construction in the training set and evaluation in the test set

**FIGURE 2:**
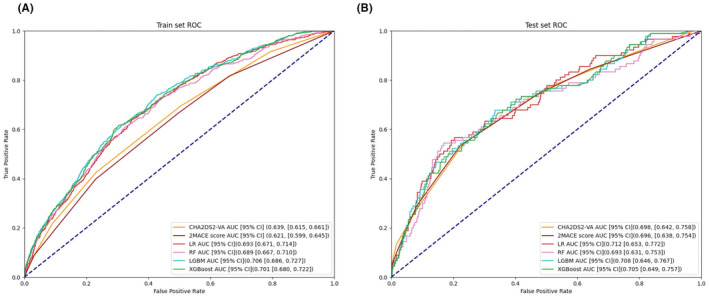
ROC curves of prediction models in train set (A) and test set (B). AUC, areas under the ROC curve; CI, confidence interval; LGBM, Light Gradient Boosting Machine; LR, Logistic Regression; RF, Random Forest; ROC, Receiver operating characteristic; XGBM, Extreme Gradient Boosting Machin.

We use several metrics to estimate the performance of ML models, shown in Table 2, and the ROC curves in Figure 2.

**TABLE 2:** Metrics to estimate models' performance.

	LR	RF	LGBM	XGBM	2MACE score	CHA_2_DS_2_‐VA score
Train set						
G mean	0.210	0.206	0.218	0.214	0.184	0.191
F1 Score	0.124	0.125	0.119	0.134	0.095	0.097
Accuracy	0.652	0.673	0.577	0.694	0.509	0.503
Precision	0.069	0.070	0.064	0.075	0.051	0.052
Recall	0.639	0.608	0.737	0.612	0.666	0.695
Specificity	0.653	0.675	0.571	0.697	0.503	0.495
AUC [95% CI]	0.693 [0.671, 0.714]	0.689 [0.667, 0.710]	0.706 [0.686, 0.727]	0.701 [0.680, 0.722]	0.621 [0.599, 0.645]	0.639 [0.615, 0.661]
*p* value^a^	<0.001	<0.001	<0.001	<0.001	0.023	Reference
Test set						
G mean	0.283	0.295	0.269	0.265	0.256	0.258
F1 Score	0.229	0.249	0.187	0.184	0.158	0.156
Accuracy	0.782	0.813	0.663	0.661	0.538	0.517
Precision	0.144	0.163	0.109	0.107	0.088	0.087
Recall	0.556	0.533	0.667	0.656	0.744	0.767
Specificity	0.796	0.831	0.663	0.662	0.525	0.767
AUC [95% CI]	0.712 [0.653, 0.772]	0.693 [0.631, 0.772]	0.708 [0.647, 0.764]	0.705 [0.646, 0.767]	0.696 [0.642, 0.758]	0.698 [0.638, 0.754]
*p* value^a^	0.500	0.852	0.637	0.742	0.923	Reference

^a^ Delong tests were used to compare AUC.

Abbreviations: AUC, areas under the receiver operating characteristic curve; CI: confidence interval; LR, Logistic Regression; RF, Random Forest; LGBM, Light Gradient Boosting Machine; XGBM, Extreme Gradient Boosting Machin.

In the train set, LGBM has the highest g‐means of 0.218, with an F1 score of 0.119 and an AUC of 0.706 (95% CI 0.686–0.727, *p* < 0.001). All ML models had higher AUC than the CHA_2_DS_2_‐VA score and 2MACE score (*p* < 0.001).

In the test set, LR has the best performance with the higher trend of AUC [0.712 (95% CI 0.653–0.772, *p* = 0.500)]. RF have highest g‐means of 0.295, followed by the highest F1 score of 0.249 and an AUC of 0.693 (95% CI 0.631–0.772, *p* = 0.852). ML models were superior to the performance of the CHA_2_DS_2_‐VA score and 2MACE score, where the G‐mean and F1 scores were lower.

### 3.4 Sensitivity analysis

To eliminate the impact of follow‐up duration, we excluded patients with less than one year of follow‐up and assessed the performance of the ML models. The results are presented in Table S2, with the corresponding ROC curves shown in Figure S3. In the train set, ML models had better performance than CHA_2_DS_2_‐VA score and 2MACE score. In the test set, ML models had better performance than traditional clinical scores with higher AUC and higher F1 score. LR has the best performance with the higher AUC of 0.718 (95% CI 0.661–0.769, *p* < 0.001). Sensitivity analysis is consistent with the main analysis.

### 3.5 Feature importance

**FIGURE 3:**
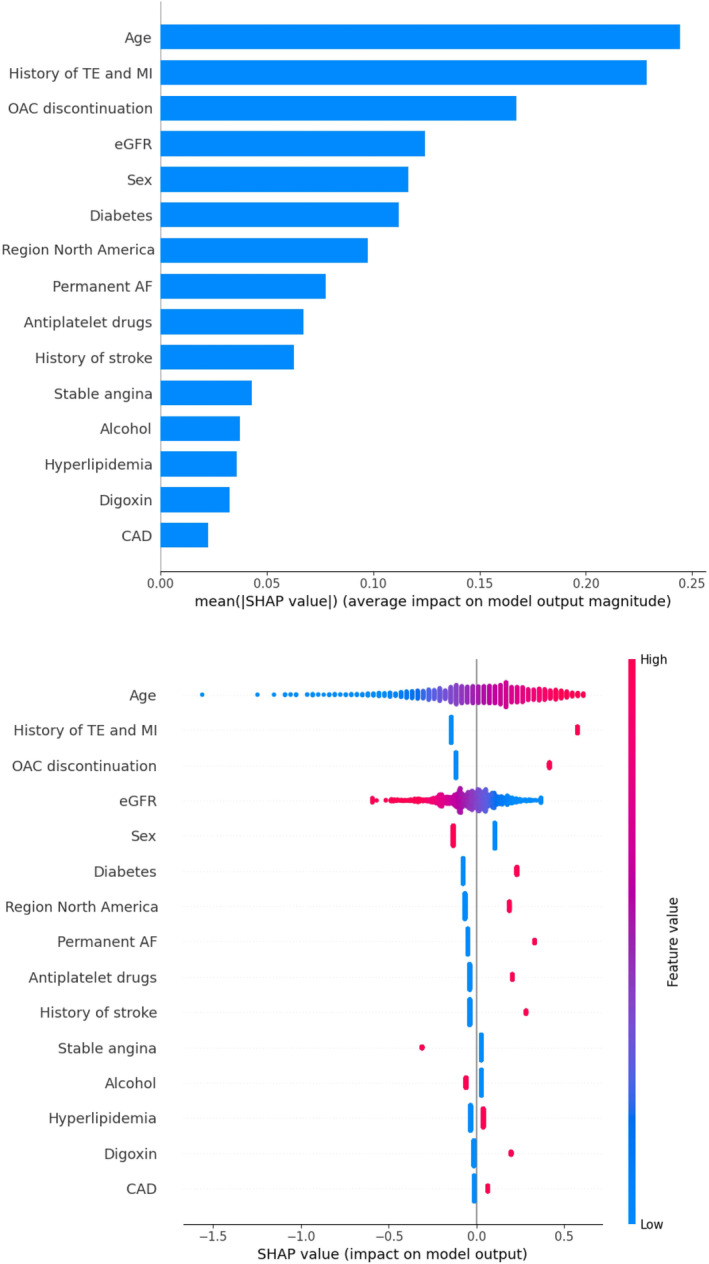
SHAP plot and of LGBM model (A) The importance ranking of the top 20 variables according to the mean (|SHAP value|) in LGBM; (B) The importance ranking of the top 20 variables with stability and interpretation using the optimal model in LGBM. The higher SHAP value of a feature is given, the higher risk of residual risk the patient would have. The red part in feature value represents a higher value. CAD, coronary artery disease; LGBM, Light Gradient Boosting Machine; estimated Glomerular Filtration Rate; NOAC, Non‐vitamin K antagonist oral anticoagulant; SHAP, SHapley Additive exPlanations; VKA, vitamin K antagonist.

Figure 3 shows feature importance evaluated by the average absolute SHAP value in the LR model. In each feature‐important row in Figure 3B, the red dots represent high risk while the blue dots represent low risk. The results show the top five important features, including age, history of TE or MI, OAC discontinuation eGFR and sex, were associated with residual risk.

## 4 DISCUSSION

This study developed four ML models to predict the residual risk of thrombotic events in anticoagulated AF patients, based on patients enrolled in the global, prospective GLORIA‐AF registry. Our findings show that the ML models outperformed traditional clinical risk scores including the CHA_2_DS_2_‐VA score and 2MACE score.

The enhanced accuracy of our ML models could be attributed to the incorporation of additional risk factors and continuous variables (eGFR) that were not considered in the conventional clinical risk scoring systems. Feature importance ranking showed that age, history of TE or MI, OAC discontinuation, eGFR and sex were associated with the composite residual thrombotic risk in anticoagulated AF patients.

Compared with previous randomized controlled trials,^35^, ^36^, ^37^ our study has a lower incidence rate of thrombotic risk, which might be attributed to the characteristics of our patients, who exhibit fewer comorbidities, better anticoagulation management, and a clinical trial medical setting.^19^ In a recent study involving populations from five pivotal randomized trials of antithrombotic therapy in AF, 35.4% of patients had a history of stroke before trial enrolment, and following a follow‐up period of 337 (102–617) days, 74 patients (6.4%) experienced a recurrent ischemic stroke.^38^

Our study also found that AF patients with residual risk were older and had higher CHA_2_DS_2_‐VA scores and more comorbidities, consistent with prior evidence.^37^

The CHA_2_DS_2_‐VA score has been established as a reliable, simple clinical tool for prognostic assessment in AF patients for many years as part of guideline‐directed medical therapy,^27^, ^39^ while the 2MACE score was specifically developed as a risk‐stratification tool to predict cardiovascular outcomes in AF.^30^ A previous study from the GLORIA‐AF registry reported that the 2MACE could predict major adverse cardiovascular events, including MI, stroke, and cardiovascular death in AF patients.^20^

Our study shows that while traditional clinical scores may offer some insights into assessing residual thrombotic event risk. However, their ability to accurately predict positive cases is limited, as reflected by the lower F1 score and recall. Other calculators or models can be used to estimate residual risk after anticoagulation. For example, Ding et al. studied a modified CARS to predict the residual risk of one‐year stroke.^40^ The modified CARS (mCARS) was calculated by multiplying the calculated CARS^41^ by 0.36, and the AUC of mCARs was 0.678 (95% CI, 0.598–0.758) in the clinical trial and 0.712 (95% CI, 0.618–0.805) in a real‐world cohort, respectively. However, there's a limited number of models or scores to predict residual risk.

In clinical practice, the CHA_2_DS_2_‐VA score has demonstrated superior practicality and generalizability, having been validated by multiple studies.^27^, ^39^, ^42^ ML models, while powerful in data mining and feature discovery, are not as easily calculated as traditional scores, which limits their broader application. However, the ability of ML models to identify novel risk factors not considered in traditional models provides significant value for future model development and disease mechanism research, especially in the context of ever‐evolving and multidimensional clinical data. Additionally, their nonlinear processing capabilities offer the potential for more personalized risk predictions, particularly for complex patient populations.^43^ While ML models may face challenges in current applications, they hold considerable promise for future research and clinical practice. In addition, traditional risk stratification models have many limitations. For example, the CHA_2_DS_2_‐VA score probably overlooks the potential residual risk that may persist despite anticoagulation.^4^, ^40^ Our ML models were able to identify and incorporate a wider range of risk factors, thereby improving the prediction of residual risk. Therefore, there is a need for more comprehensive variables and advanced algorithms to significantly improve their predictive performance, a limitation that should be addressed in future studies.

According to SHAP values, our study demonstrated some common risk factors relative to residual risk, including age, history of TE or MI, OAC discontinuation, eGFR and sex. Several previous studies have similar results to our findings. For example, Ding et al. demonstrated that residual risk of ischemic stroke whilst on anticoagulant therapy was associated with age (HR 1.05 [95% CI, 1.03–1.07]), diabetes (HR 1.42 [95% CI, 1.08–1.87]), and prior TE (HR 2.27 [95% CI, 1.73–2.98]).^19^ A real‐world study from Japan also found that older age (65–74 vs. <65 years; adjusted HR 2.02 (95% CI 1.49 to 2.73)) and hypertension (adjusted HR 1.41 (1.04 to 1.92)) significantly increased the risks of ischaemic stroke and systemic embolism.^44^ Similar results were also found in a post‐hoc analysis of the SPORTIF III and V trials, in which older age (>75 years), previous stroke/TIA, coronary artery disease and smoking have a significantly increased risk of IS/SE.^45^ In the study by Chen et al., age was also considered an important feature in an RF model predicting stroke in Asian patients with AF, with AUC 0.821 (95% CI 0.816–0.825) in the internal validation cohort.^21^

The present study also found that eGFR is a significant feature in the ML model prediction. Chronic kidney disease independently increases the risk of thromboembolism in AF.^46^ The efficacy and safety of OAC therapy varies across different eGFR levels,^47^ probably attributed to variations in renal elimination.^48^ Moderate (creatinine clearance [CrCl] 50–80 mL/min) and severe (CrCl <50 mL/min) renal impairment was significantly associated with an increased risk of IS/SE with adjusted HR of 1.54 and 2.22, respectively.^6^

Coronary artery disease and hyperlipidaemia were two important features included in the ML models. Patients that occurred residual risk also have higher prevalence of coronary artery disease and hyperlipidaemia. Despite proper anticoagulation, residual ischemic events may still occur due to a combination of embolic events and local atherosclerosis. For instance, patients with a higher burden of atherosclerotic disease may experience more events related to plaque rupture or progression,^49^ whereas those with higher embolic potential may suffer more embolic complications despite therapeutic anticoagulation.

### 4.1 Strengths and limitations

The main strengths of our study can be summarized: First, the number of studies that look at the prediction of residual risk in AF patients with OAC therapy is limited, and this study provides innovative approaches to assess the residual risk in these anticoagulated patients. Moreover, leveraging the rich data on AF patients who received OAC therapy (over 80%) in the GLORIA‐AF Registry,^50^ ML algorithms present an opportunity to develop more precise and personalized risk prediction models for AF patients on OAC therapy. Second, the residual risk is multifactorial, and ML algorithms can consider multiple factors affecting outcomes to provide predictions, in addition to relative risk factors that are not included in current estimated calculators, for example, eGFR, antiplatelet drugs, and type of AF. Also, ML models can consider the potential impact of continuous variations on residual risk, other than categorical variables in traditional scores, such as age, blood pressure, and BMI. Thus, our models provide a more comprehensive evaluation of residual risk following OAC treatment. This may facilitate the use of more holistic or integrated care approaches to AF management, which has been shown to improve clinical outcomes.^51^, ^52^

There are also some limitations in our study. First, the incidence rate of positive events was low, around 4.0%, resulting in an imbalanced training set. Although we attempted feature selection, weighted distribution during model construction, and evaluated metrics to mitigate the effects of unbalanced data, the impact persisted. Second, our model lacks external validation, although the selected variables mostly consist of clinically common indicators, which should have relatively robust applicability. In addition, our study had no data on other potential risk features, such as cardiac surgery, laboratory biomarkers (e.g. troponin),^53^, ^54^ electrocardiograms^55^ and echocardiograms,^56^ including relevant variables like left atrial size. We also lacked data of OAC intake in detailed, including OAC dosage. The registry data did not differentiate between ischemic stroke subtypes, such as cardioembolic or other causes, which may affect the interpretation of responses to interventions. Future studies should include a more detailed analysis of stroke subtypes. Furthermore, anticoagulant use is influenced by other factors such as physician expertise, patient adherence, treatment strategies, and potential interactions with other medications, all of which might affect outcomes associated with residual risk.

## 5 CONCLUSION

In this study, we developed four ML models—LR, RF, LGBM and XGBM—to predict residual thrombotic risk in AF patients receiving anticoagulation therapy, using data from the GLORIA‐AF registry. Notably, the LR model demonstrated the highest AUC, while the RF model had the highest g‐means and F1 score, indicating these models' relatively better performance. Although the AUC improvements were modest compared to the CHA_2_DS_2_‐VA and 2MACE scores, the observed increase highlights the additional insights provided by the ML algorithms. Furthermore, age, history of TE or MI, OAC discontinuation, eGFR and sex were identified as the top five influential features in the LR model, underscoring their significance in predicting residual thrombotic risk.

## AUTHOR CONTRIBUTIONS

YL, YC and GYHL designed the study. YL did the analysis and wrote the draft with YC who contributed to the interpretation of the data. IO and SOM contributed to statistical methods. IO, SOM, BH, HI, HML, KH, MVH and GYHL contributed to substantive revision. All authors read and approved the final manuscript. GYHL is the guarantor of this paper.

## FUNDING INFORMATION

This study was funded by Boehringer Ingelheim GmbH support for the GLORIA‐AF registry and Nanchang University Abroad Scholarship, who funded Dr. Yang Liu. The authors are solely responsible for the design and conduct of this study, all study analyses, the drafting and editing of the manuscript, and its final contents.

## CONFLICT OF INTEREST STATEMENT

YL was an honorary associate research fellow at the University of Liverpool and funded by Professor Kui Hong's national key research and development project in the Second Hospital of Nanchang University and Nanchang University Abroad Scholarship. MVH reports receiving research grants from the Dutch Healthcare Fund, Dutch Heart Foundation, BMS‐Pfizer, Bayer Healthcare and Boehringer Ingelheim and consulting fees from BMS‐Pfizer, Bayer Healthcare and Boehringer Ingelheim to the institution. GYHL is the consultant and speaker for BMS/Pfizer, Boehringer Ingelheim, Daiichi‐Sankyo, and Anthos. No fees are received personally. He is a National Institute for Health and Care Research (NIHR) Senior Investigator and co‐PI of the AFFIRMO project on multimorbidity in AF (grant agreement No 899871), TARGET project on digital twins for personalized management of atrial fibrillation and stroke (grant agreement No 101136244) and ARISTOTELES project on artificial intelligence for management of chronic long‐term conditions (grant agreement No 101080189), which are all funded by the EU's Horizon Europe Research & Innovation programme. SOM is the PI of the TARGET project and senior investigator in the ARISTOTELES project. IO is co‐lead in TARGET and partner lead in ARISTOTELES. Other authors declare no competing interests.

## Supporting information


Appendix S1.

## Data Availability

Data supporting this study by the data contributors Boehringer Ingelheim and were made and are available through Vivli, Inc. Access was provided after a proposal was approved by an independent review committee identified for this purpose and after receipt of a signed data sharing agreement.
