# Supplementary material for: Residual risk prediction in anticoagulated patients with atrial fibrillation using machine learning: A report from the GLORIA‐AF registry phase II/III
Source: Eur J Clin Invest. 2024 Dec 11;55(3):e14371. doi: 10.1111/eci.14371 (PMC11810544; doi:10.1111/eci.14371)
Supplement: Supplementary file 1 — Appendix S1. [file ECI-55-e14371-s001.docx]

**Residual Risk Prediction in Anticoagulated Patients with Atrial Fibrillation using Machine Learning: A Report from the GLORIA-AF Registry Phase II/III**

Yang Liu^1,2#^, Yang Chen^1#^, Ivan Olier^1,3^, Sandra Ortega-Martorell^1,3^, Bi Huang^1,4^, Hironori Ishiguchi^1,5^, Ho Man Lam^1^, Kui Hong^2,6,7^, Menno V. Huisman ^8†^, Gregory Y. H. Lip^1,9 *†^

*on behalf of the GLORIA-AF Investigators*

**Supplementary materials**

[**Appendix – List of GLORIA-AF Investigators** 2](#_Toc180136395)

[**Supplementary figure 1: Spearman coefficient plot of all variables** 8](#_Toc180136396)

[**Supplementary figure 2: Spearman coefficient plot (A) and VIF score plot (B) of selected features** 9](#_Toc180136397)

[**Supplementary figure 3: Sensitivity analysis: ROC curves of prediction models in train set (A) and test set (B) in patients with over one year follow-up**. 9](#_Toc180136398)

[**Supplementary table 1: Baseline characteristic in train and test set** 10](#_Toc180136399)

[**Supplementary table 2: Sensitivity analysis: Metrics to estimate models’ performance in patients with over one year follow-up.** 11](#_Toc180136400)

# Appendix – List of GLORIA-AF Investigators

| Dzifa Wosornu Abban | Bouziane Benhalima | Jei Keon Chae |
| --- | --- | --- |
| Nasser Abdul | Jutta Bergler-Klein | Kathrine Chalamidas |
| Atilio Marcelo Abud | Jean-Baptiste Berneau | Krishnan Challappa |
| Fran Adams | Richard A. Bernstein | Sunil Prakash Chand |
| Srinivas Addala | Percy Berrospi | Harinath Chandrashekar |
| Pedro Adragão | Sergio Berti | Ludovic Chartier |
| Walter Ageno | Andrea Berz | Kausik Chatterjee |
| Rajesh Aggarwal | Elizabeth Best | Carlos Antero Chavez Ayala |
| Sergio Agosti | Paulo Bettencourt | Aamir Cheema |
| Piergiuseppe Agostoni | Robert Betzu | Amjad Cheema |
| Francisco Aguilar | Ravi Bhagwat | Lin Chen |
| Julio Aguilar Linares | Luna Bhatta | Shih-Ann Chen |
| Luis Aguinaga | Francesco Biscione | Jyh Hong Chen |
| Jameel Ahmed | Giovanni Bisignani | Fu-Tien Chiang |
| Allessandro Aiello | Toby Black | Francesco Chiarella |
| Paul Ainsworth | Michael J. Bloch | Lin Chih-Chan |
| Jorge Roberto Aiub | Stephen Bloom | Yong Keun Cho |
| Raed Al-Dallow | Edwin Blumberg | Jong-Il Choi |
| Lisa Alderson | Mario Bo | Dong Ju Choi |
| Jorge Antonio Aldrete Velasco | Ellen Bøhmer | Guy Chouinard |
| Dimitrios Alexopoulos | Andreas Bollmann | Danny Hoi-Fan Chow |
| Fernando Alfonso Manterola | Maria Grazia Bongiorni | Dimitrios Chrysos |
| Pareed Aliyar | Giuseppe Boriani | Galina Chumakova |
| David Alonso | D.J. Boswijk | Eduardo Julián José Roberto Chuquiure Valenzuela |
| Fernando Augusto Alves da Costa | Jochen Bott | Nicoleta Cindea Nica |
| José Amado | Edo Bottacchi | David J. Cislowski |
| Walid Amara | Marica Bracic Kalan | Anthony Clay |
| Mathieu Amelot | Drew Bradman | Piers Clifford |
| Nima Amjadi | Donald Brautigam | Andrew Cohen |
| Fabrizio Ammirati | Nicolas Breton | Michael Cohen |
| Marianna Andrade | P.J.A.M. Brouwers | Serge Cohen |
| Nabil Andrawis | Kevin Browne | Furio Colivicchi |
| Giorgio Annoni | Jordi Bruguera Cortada | Ronan Collins |
| Gerardo Ansalone | A. Bruni | Paolo Colonna |
| M.Kevin Ariani | Claude Brunschwig | Steve Compton |
| Juan Carlos Arias | Hervé Buathier | Derek Connolly |
| Sébastien Armero | Aurélie Buhl | Alberto Conti |
| Chander Arora | John Bullinga | Gabriel Contreras Buenostro |
| Muhammad Shakil Aslam | Jose Walter Cabrera | Gregg Coodley |
| M. Asselman | Alberto Caccavo | Martin Cooper |
| Philippe Audouin | Shanglang Cai | Julian Coronel |
| Charles Augenbraun | Sarah Caine | Giovanni Corso |
| S. Aydin | Leonardo Calò | Juan Cosín Sales |
| Ivaneta Ayryanova | Valeria Calvi | Yves Cottin |
| Emad Aziz | Mauricio Camarillo Sánchez | John Covalesky |
| Luciano Marcelo Backes | Rui Candeias | Aurel Cracan |
| E. Badings | Vincenzo Capuano | Filippo Crea |
| Ermentina Bagni | Alessandro Capucci | Peter Crean |
| Seth H. Baker | Ronald Caputo | James Crenshaw |
| Richard Bala | Tatiana Cárdenas Rizo | Tina Cullen |
| Antonio Baldi | Francisco Cardona | Harald Darius |
| Shigenobu Bando | Francisco Carlos da Costa Darrieux | Patrick Dary |
| Subhash Banerjee | Yan Carlos Duarte Vera | Olivier Dascotte |
| Alan Bank | Antonio Carolei | Ira Dauber |
| Gonzalo Barón Esquivias | Susana Carreño | Vicente Davalos |
| Craig Barr | Paula Carvalho | Ruth Davies |
| Maria Bartlett | Susanna Cary | Gershan Davis |
| Vanja Basic Kes | Gavino Casu | Jean-Marc Davy |
| Giovanni Baula | Claudio Cavallini | Mark Dayer |
| Steffen Behrens | Guillaume Cayla | Marzia De Biasio |
| Alan Bell | Aldo Celentano | Silvana De Bonis |
| Raffaella Benedetti | Tae-Joon Cha | Raffaele De Caterina |
| Juan Benezet Mazuecos | Kwang Soo Cha | Teresiano De Franceschi |
| J.R. de Groot | William French | Christian Hall |
| José De Horta | Keith Friedman | Bing Han |
| Axel De La Briolle | Athena Friese | Seongwook Han |
| Gilberto de la Pena Topete | Ana Gabriela Fruntelata | Joe Hargrove |
| Angelo Amato Vicenzo de Paola | Shigeru Fujii | David Hargroves |
| Weimar de Souza | Stefano Fumagalli | Kenneth B. Harris |
| A. de Veer | Marta Fundamenski | Tetsuya Haruna |
| Luc De Wolf | Yutaka Furukawa | Emil Hayek |
| Eric Decoulx | Matthias Gabelmann | Jeff Healey |
| Sasalu Deepak | Nashwa Gabra | Steven Hearne |
| Pascal Defaye | Niels Gadsbøll | Michael Heffernan |
| Freddy Del-Carpio Munoz | Michel Galinier | Geir Heggelund |
| Diana Delic Brkljacic | Anders Gammelgaard | J.A. Heijmeriks |
| N. Joseph Deumite | Priya Ganeshkumar | Maarten Hemels |
| Silvia Di Legge | Christopher Gans | I. Hendriks |
| Igor Diemberger | Antonio Garcia Quintana | Sam Henein |
| Denise Dietz | Olivier Gartenlaub | Sung-Ho Her |
| Pedro Dionísio | Achille Gaspardone | Paul Hermany |
| Qiang Dong | Conrad Genz | Jorge Eduardo Hernández Del Río |
| Fabio Rossi dos Santos | Frédéric Georger | Yorihiko Higashino |
| Elena Dotcheva | Jean-Louis Georges | Michael Hill |
| Rami Doukky | Steven Georgeson | Tetsuo Hisadome |
| Anthony D'Souza | Evaldas Giedrimas | Eiji Hishida |
| Simon Dubrey | Mariusz Gierba | Etienne Hoffer |
| Xavier Ducrocq | Ignacio Gil Ortega | Matthew Hoghton |
| Dmitry Dupljakov | Eve Gillespie | Kui Hong |
| Mauricio Duque | Alberto Giniger | Suk keun Hong |
| Dipankar Dutta | Michael C. Giudici | Stevie Horbach |
| Nathalie Duvilla | Alexandros Gkotsis | Masataka Horiuchi |
| A. Duygun | Taya V. Glotzer | Yinglong Hou |
| Rainer Dziewas | Joachim Gmehling | Jeff Hsing |
| Charles B. Eaton | Jacek Gniot | Chi-Hung Huang |
| William Eaves | Peter Goethals | David Huckins |
| L.A Ebels-Tuinbeek | Seth Goldbarg | kathy Hughes |
| Clifford Ehrlich | Ronald Goldberg | A. Huizinga |
| Sabine Eichinger-Hasenauer | Britta Goldmann | E.L. Hulsman |
| Steven J. Eisenberg | Sergey Golitsyn | Kuo-Chun Hung |
| Adnan El Jabali | Silvia Gómez | Gyo-Seung Hwang |
| Mahfouz El Shahawy | Juan Gomez Mesa | Margaret Ikpoh |
| Mauro Esteves Hernandes | Vicente Bertomeu Gonzalez | Davide Imberti |
| Ana Etxeberria Izal | Jesus Antonio Gonzalez Hermosillo | Hüseyin Ince |
| Rudolph Evonich III | Víctor Manuel González López | Ciro Indolfi |
| Oksana Evseeva | Hervé Gorka | Shujiro Inoue |
| Andrey Ezhov | Charles Gornick | Didier Irles |
| Raed Fahmy | Diana Gorog | Harukazu Iseki |
| Quan Fang | Venkat Gottipaty | C. Noah Israel |
| Ramin Farsad | Pascal Goube | Bruce Iteld |
| Laurent Fauchier | Ioannis Goudevenos | Venkat Iyer |
| Stefano Favale | Brett Graham | Ewart Jackson-Voyzey |
| Maxime Fayard | G. Stephen Greer | Naseem Jaffrani |
| Jose Luis Fedele | Uwe Gremmler | Frank Jäger |
| Francesco Fedele | Paul G. Grena | Martin James |
| Olga Fedorishina | Martin Grond | Sung-Won Jang |
| Steven R. Fera | Edoardo Gronda | Nicolas Jaramillo |
| Luis Gustavo Gomes Ferreira | Gerian Grönefeld | Nabil Jarmukli |
| Jorge Ferreira | Xiang Gu | Robert J. Jeanfreau |
| Claudio Ferri | Ivett Guadalupe Torres Torres | Ronald D. Jenkins |
| Anna Ferrier | Gabriele Guardigli | Carlos Jerjes Sánchez |
| Hugo Ferro | Carolina Guevara | Javier Jimenez |
| Alexandra Finsen | Alexandre Guignier | Robert Jobe |
| Brian First | Michele Gulizia | Tomas Joen-Jakobsen |
| Stuart Fischer | Michael Gumbley | Nicholas Jones |
| Catarina Fonseca | Albrecht Günther | Jose Carlos Moura Jorge |
| Luísa Fonseca Almeida | Andrew Ha | Bernard Jouve |
| Steven Forman | Georgios Hahalis | Byung Chun Jung |
| Brad Frandsen | Joseph Hakas | Kyung Tae Jung |
| Werner Jung | Torben Larsen | Nolan Mayer |
| Mikhail Kachkovskiy | Karine Lavandier | John McClure |
| Krystallenia Kafkala | Jessica LeBlanc | Terry McCormack |
| Larisa Kalinina | Moon Hyoung Lee | William McGarity |
| Bernd Kallmünzer | Chang-Hoon Lee | Hugh McIntyre |
| Farzan Kamali | John Lehman | Brent McLaurin |
| Takehiro Kamo | Ana Leitão | Feliz Alvaro Medina Palomino |
| Priit Kampus | Nicolas Lellouche | Francesco Melandri |
| Hisham Kashou | Malgorzata Lelonek | Hiroshi Meno |
| Andreas Kastrup | Radoslaw Lenarczyk | Dhananjai Menzies |
| Apostolos Katsivas | T. Lenderink | Marco Mercader |
| Elizabeth Kaufman | Salvador León González | Christian Meyer |
| Kazuya Kawai | Peter Leong-Sit | Beat j. Meyer |
| Kenji Kawajiri | Matthias Leschke | Jacek Miarka |
| John F. Kazmierski | Nicolas Ley | Frank Mibach |
| P Keeling | Zhanquan Li | Dominik Michalski |
| José Francisco Kerr Saraiva | Xiaodong Li | Patrik Michel |
| Galina Ketova | Weihua Li | Rami Mihail Chreih |
| AJIT Singh Khaira | Xiaoming Li | Ghiath Mikdadi |
| Aleksey Khripun | Christhoh Lichy | Milan Mikus |
| Doo-Il Kim | Ira Lieber | Davor Milicic |
| Young Hoon Kim | Ramon Horacio Limon Rodriguez | Constantin Militaru |
| Nam Ho Kim | Hailong Lin | Sedi Minaie |
| Dae Kyeong Kim | Gregory Y. H. Lip | Bogdan Minescu |
| Jeong Su Kim | Feng Liu | Iveta Mintale |
| June Soo Kim | Hengliang Liu | Tristan Mirault |
| Ki Seok Kim | Guillermo Llamas Esperon | Michael J. Mirro |
| Jin bae Kim | Nassip Llerena Navarro | Dinesh Mistry |
| Elena Kinova | Eric Lo | Nicoleta Violeta Miu |
| Alexander Klein | Sergiy Lokshyn | Naomasa Miyamoto |
| James J. Kmetzo | Amador López | Tiziano Moccetti |
| G. Larsen Kneller | José Luís López-Sendón | Akber Mohammed |
| Aleksandar Knezevic | Adalberto Menezes Lorga Filho | Azlisham Mohd Nor |
| Su Mei Angela Koh | Richard S. Lorraine | Michael Mollerus |
| Shunichi Koide | Carlos Alberto Luengas | Giulio Molon |
| Anastasios Kollias | Robert Luke | Sergio Mondillo |
| J.A. Kooistra | Ming Luo | Patrícia Moniz |
| Jay Koons | Steven Lupovitch | Lluis Mont |
| Martin Koschutnik | Philippe Lyrer | Vicente Montagud |
| William J. Kostis | Changsheng Ma | Oscar Montaña |
| Dragan Kovacic | Genshan Ma | Cristina Monti |
| Jacek Kowalczyk | Irene Madariaga | Luciano Moretti |
| Natalya Koziolova | Koji Maeno | Kiyoo Mori |
| Peter Kraft | Dominique Magnin | Andrew Moriarty |
| Johannes A. Kragten | Gustavo Maid | Jacek Morka |
| Mori Krantz | Sumeet K. Mainigi | Luigi Moschini |
| Lars Krause | Konstantinos Makaritsis | Nikitas Moschos |
| B.J. Krenning | Rohit Malhotra | Andreas Mügge |
| F. Krikke | Rickey Manning | Thomas J. Mulhearn |
| Z. Kromhout | Athanasios Manolis | Carmen Muresan |
| Waldemar Krysiak | Helard Andres Manrique Hurtado | Michela Muriago |
| Priya Kumar | Ioannis Mantas | Wlodzimierz Musial |
| Thomas Kümler | Fernando Manzur Jattin | Carl W. Musser |
| Malte Kuniss | Vicky Maqueda | Francesco Musumeci |
| Jen-Yuan Kuo | Niccolo Marchionni | Thuraia Nageh |
| Achim Küppers | Francisco Marin Ortuno | Hidemitsu Nakagawa |
| Karla Kurrelmeyer | Antonio Martín Santana | Yuichiro Nakamura |
| Choong Hwan Kwak | Jorge Martinez | Toru Nakayama |
| Bénédicte Laboulle | Petra Maskova | Gi-Byoung Nam |
| Arthur Labovitz | Norberto Matadamas Hernandez | Michele Nanna |
| Wen Ter Lai | Katsuhiro Matsuda | Indira Natarajan |
| Andy Lam | Tillmann Maurer | Hemal M. Nayak |
| Yat Yin Lam | Ciro Mauro | Stefan Naydenov |
| Fernando Lanas Zanetti | Erik May | Jurica Nazlić |
| Charles Landau | Torben Larsen | Alexandru Cristian Nechita |
| Giancarlo Landini | Karine Lavandier | Libor Nechvatal |
| Estêvão Lanna Figueiredo | Jessica LeBlanc | Sandra Adela Negron |
| James Neiman | Arnold Pinter | Hamdi Sati |
| Fernando Carvalho Neuenschwander | Fausto Pinto | Irina Savelieva |
| David Neves | R. Pisters | Pierre-Jean Scala |
| Anna Neykova | Nediljko Pivac | Peter Schellinger |
| Ricardo Nicolás Miguel | Darko Pocanic | Carlos Scherr |
| George Nijmeh | Cristian Podoleanu | Lisa Schmitz |
| Alexey Nizov | Alessandro Politano | Karl-Heinz Schmitz |
| Rodrigo Noronha Campos | Zdravka Poljakovic | Bettina Schmitz |
| Janko Nossan | Stewart Pollock | Teresa Schnabel |
| Tatiana Novikova | Jose Polo Garcéa | Steffen Schnupp |
| Ewa Nowalany-Kozielska | Holger Poppert | Peter Schoeniger |
| Emmanuel Nsah | Maurizio Porcu | Norbert Schön |
| Juan Carlos Nunez Fragoso | Antonio Pose Reino | Peter Schwimmbeck |
| Svetlana Nurgalieva | Neeraj Prasad | Clare Seamark |
| Dieter Nuyens | Dalton Bertolim Précoma | Greg Searles |
| Ole Nyvad | Alessandro Prelle | Karl-Heinz Seidl |
| Manuel Odin de Los Rios Ibarra | John Prodafikas | Barry Seidman |
| Philip O'Donnell | Konstantin Protasov | Jaroslaw Sek |
| Martin O'Donnell | Maurice Pye | Lakshmanan Sekaran |
| Seil Oh | Zhaohui Qiu | Carlo Serrati |
| Yong Seog Oh | Jean-Michel Quedillac | Neerav Shah |
| Dongjin Oh | Dimitar Raev | Vinay Shah |
| Gilles O'Hara | Carlos Antonio Raffo Grado | Anil Shah |
| Kostas Oikonomou | Sidiqullah Rahimi | Shujahat Shah |
| Claudia Olivares | Arturo Raisaro | Vijay Kumar Sharma |
| Richard Oliver | Bhola Rama | Louise Shaw |
| Rafael Olvera Ruiz | Ricardo Ramos | Khalid H. Sheikh |
| Christoforos Olympios | Maria Ranieri | Naruhito Shimizu |
| Anna omaszuk-Kazberuk | Nuno Raposo | Hideki Shimomura |
| Joaquín Osca Asensi | Eric Rashba | Dong-Gu Shin |
| eena Padayattil jose | Ursula Rauch-Kroehnert | Eun-Seok Shin |
| Francisco Gerardo Padilla Padilla | Ramakota Reddy | Junya Shite |
| Victoria Padilla Rios | Giulia Renda | Gerolamo Sibilio |
| Giuseppe Pajes | Shabbir Reza | Frank Silver |
| A. Shekhar Pandey | Luigi Ria | Iveta Sime |
| Gaetano Paparella | Dimitrios Richter | Tim A. Simmers |
| F Paris | Hans Rickli | Narendra Singh |
| Hyung Wook Park | Werner Rieker | Peter Siostrzonek |
| Jong Sung Park | Tomas Ripolil Vera | Didier Smadja |
| Fragkiskos Parthenakis | Luiz Eduardo Ritt | David W. Smith |
| Enrico Passamonti | Douglas Roberts | Marcelo Snitman |
| Rajesh J. Patel | Ignacio Rodriguez Briones | Dario Sobral Filho |
| Jaydutt Patel | Aldo Edwin Rodriguez Escudero | Hassan Soda |
| Mehool Patel | Carlos Rodríguez Pascual | Carl Sofley |
| Janice Patrick | Mark Roman | Adam Sokal |
| Ricardo Pavón Jimenez | Francesco Romeo | Yannie Soo Oi Yan |
| Analía Paz | E. Ronner | Rodolfo Sotolongo |
| Vittorio Pengo | Jean-Francois Roux | Olga Ferreira de Souza |
| William Pentz | Nadezda Rozkova | Jon Arne Sparby |
| Beatriz Pérez | Miroslav Rubacek | Jindrich Spinar |
| Alma Minerva Pérez Ríos | Frank Rubalcava | David Sprigings |
| Alejandro Pérez-Cabezas | Andrea M. Russo | Alex C. Spyropoulos |
| Richard Perlman | Matthieu Pierre Rutgers | Dimitrios Stakos |
| Viktor Persic | Karin Rybak | Clemens Steinwender |
| Francesco Perticone | Samir Said | Georgios Stergiou |
| Terri K. Peters | Tamotsu Sakamoto | Ian Stiell |
| Sanjiv Petkar | Abraham Salacata | Marcus Stoddard |
| Luis Felipe Pezo | Adrien Salem | Anastas Stoikov |
| Christian Pflücke | Rafael Salguero Bodes | Witold Streb |
| David N. Pham | Marco A. Saltzman | Ioannis Styliadis |
| Roland T. Phillips | Alessandro Salvioni | Guohai Su |
| Stephen Phlaum | Gregorio Sanchez Vallejo | Xi Su |
| Denis Pieters | Marcelo Sanmartín Fernández | Wanda Sudnik |
| Kai Sukles | Alberta L. Warner | Tiziana Tassinari |
| Julien Pineau | Wladmir Faustino Saporito | Ashis Tayal |
| Jens Taggeselle | Kouki Watanabe | Muzahir Tayebjee |
| Yuichiro Takagi | Jeanne Wei | J.M. ten Berg |
| Amrit Pal Singh Takhar | Christian Weimar | Dan Tesloianu |
| Angelika Tamm | Stanislav Weiner | Salem H.K. The |
| Katsumi Tanaka | Renate Weinrich | Dierk Thomas |
| Tanyanan Tanawuttiwat | Ming-Shien Wen | Serge Timsit |
| Sherman Tang | Marcus Wiemer | Tetsuya Tobaru |
| Aylmer Tang | Preben Wiggers | Andrzej R. Tomasik. |
| Giovanni Tarsi | Andreas Wilke |  |
| Emmanuel Touze | Ping Zhang |  |
| Elina Trendafilova | Jun Zhang |  |
| W. Kevin Tsai | Shui Ping Zhao |  |
| Hung Fat Tse | Yujie Zhao |  |
| Hiroshi Tsutsui | Zhichen Zhao |  |
| Tian Ming Tu | Yang Zheng |  |
| Ype Tuininga | Jing Zhou |  |
| Minang Turakhia | Sergio Zimmermann |  |
| Samir Turk | Andrea Zini |  |
| Wayne Turner | Steven Zizzo |  |
| Arnljot Tveit | Wenxia Zong |  |
| Richard Tytus | L Steven Zukerman |  |
| C Valadão | Mikhail Torosoff |  |
| P.F.M.M. van Bergen | Xingwei Zhang |  |
| Philippe van de Borne |  |  |
| B.J. van den Berg |  |  |
| C van der Zwaan |  |  |
| M. Van Eck |  |  |
| Peter Vanacker |  |  |
| Dimo Vasilev |  |  |
| Vasileios Vasilikos |  |  |
| Maxim Vasilyev |  |  |
| Srikar Veerareddy |  |  |
| Mario Vega Miño |  |  |
| Asok Venkataraman |  |  |
| Paolo Verdecchia |  |  |
| Francesco Versaci |  |  |
| Ernst Günter Vester |  |  |
| Hubert Vial |  |  |
| Jason Victory |  |  |
| Alejandro Villamil |  |  |
| Marc Vincent |  |  |
| Anthony Vlastaris |  |  |
| Jürgen vom Dahl |  |  |
| Kishor Vora |  |  |
| Robert B. Vranian |  |  |
| Paul Wakefield |  |  |
| Ningfu Wang |  |  |
| Mingsheng Wang |  |  |
| Xinhua Wang |  |  |
| Feng Wang |  |  |
| Tian Wang |  |  |
| David Williams |  |  |
| Marcus L. Williams |  |  |
| Bernhard Witzenbichler |  |  |
| Brian Wong |  |  |
| Ka Sing Lawrence Wong |  |  |
| Beata Wozakowska-Kaplon |  |  |
| Shulin Wu |  |  |
| Richard C. Wu |  |  |
| Silke Wunderlich |  |  |
| Nell Wyatt |  |  |
| John (Jack) Wylie |  |  |
| Yong Xu |  |  |
| Xiangdong Xu |  |  |
| Hiroki Yamanoue |  |  |
| Takeshi Yamashita |  |  |
| Ping Yen Bryan Yan |  |  |
| Tianlun Yang |  |  |
| Yoto Yotov |  |  |
| Ralf Zahn |  |  |
| Stuart Zarich |  |  |
| Sergei Zenin |  |  |
| Elisabeth Louise Zeuthen |  |  |
| Huanyi Zhang |  |  |
| Donghui Zhang |  |  |


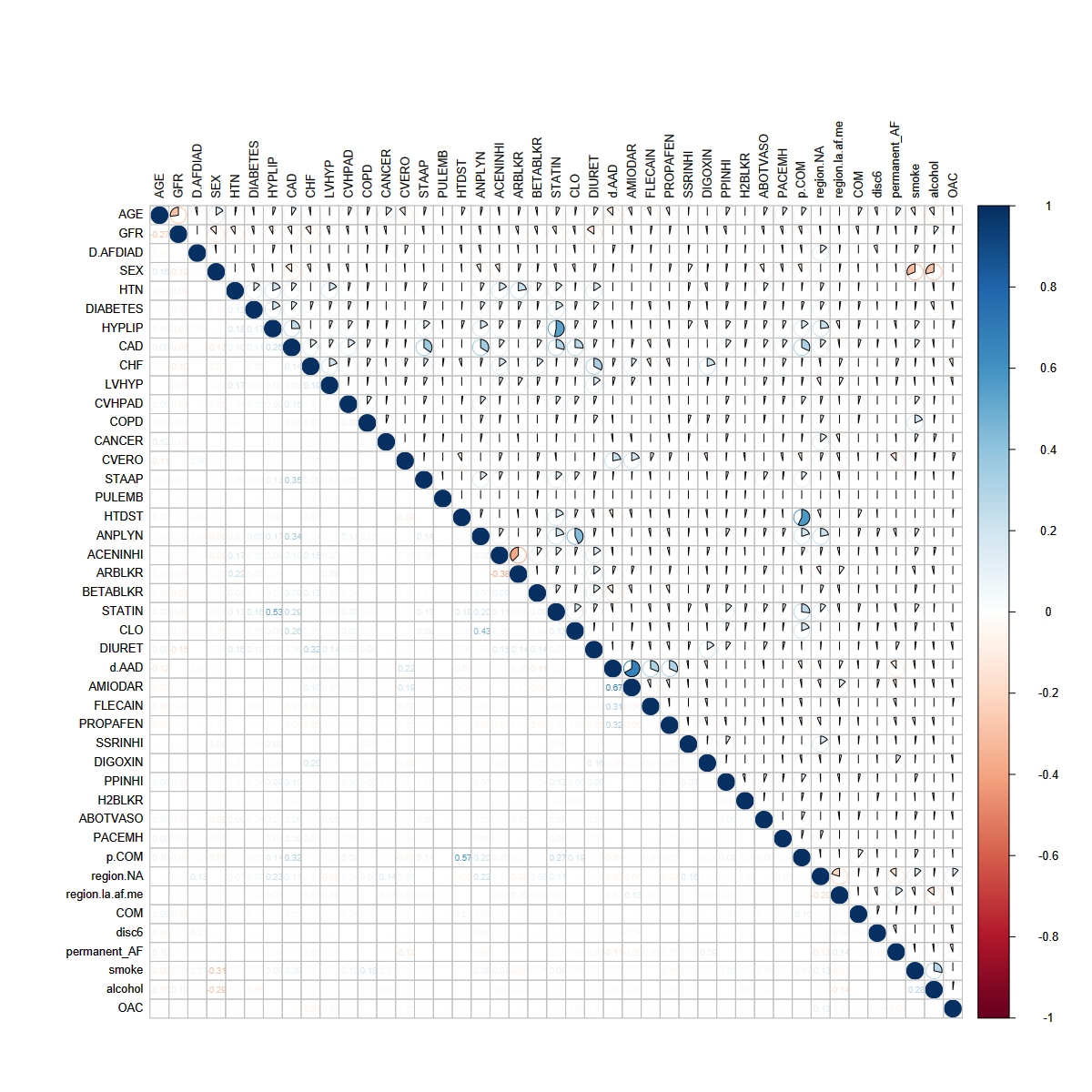


# Supplementary figure 1: Spearman coefficient plot of all variables

p.COM: history of MI and TE; GFR: Glomerular filtration rate; HTDST: previous stroke; HTN: Hypertension; HYPLIP: Hyperlipidemia; CAD: coronary artery disease; CHF: congestive heart failure; LVHYP: Left ventricular hypertrophy; CVHPAD: peripheral artery disease; COPD: chronic obstructive pulmonary disease; CVERO: previous cardioversion; STAAP: stable angina; PULEMB: Previous pulmonary embolism; HTDST: previous stroke; ANPLYN: antiplatelet drugs; ACEI angiotensin-converting enzyme inhibitors; ARB: angiotensin II receptor blockers; CLO: clopidogrel use; d.AAD: antiarrhythmic drugs use; AMIODAR: amiodarone use; FLECAIN: Flecainide use; PROPAFEN: propafenone use; SSRINHI: Selective Serotonin Reuptake Inhibitor use; H2BLKR: H2 blocker; ABOTVASO: alpha blocker or other vasodilator use; PACEMH: pacemaker in situ; COM: residual risk; OAC: oral anticoagulants use (NOAC *vs.* VKA); PPINHI: Proton pump inhibitor use; NOAC: Non-vitamin K antagonist oral anticoagulant; VKA: vitamin K antagonist;


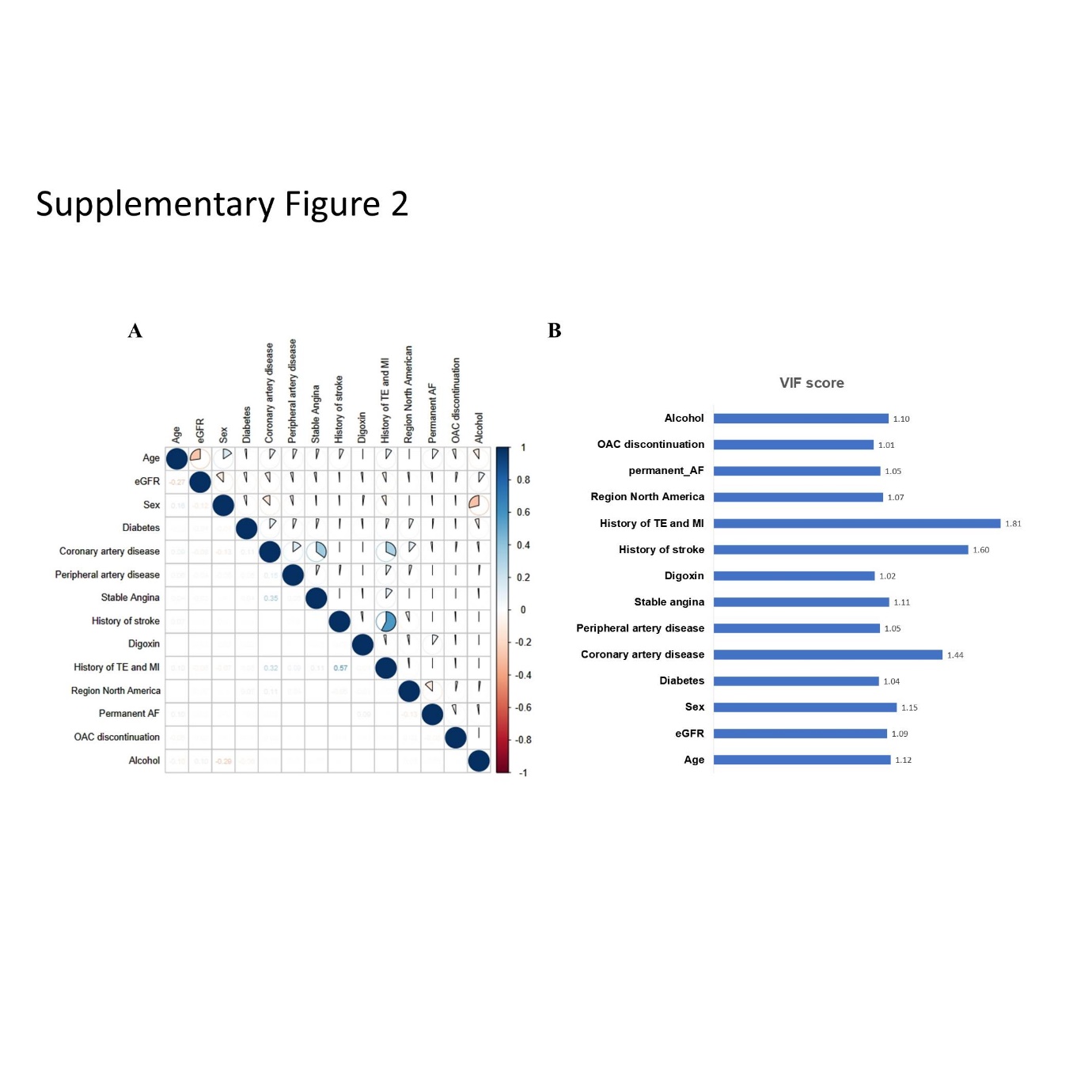


# Supplementary figure 2: Spearman coefficient plot (A) and VIF score plot (B) of selected features

VIF: Variance Inflation Factor


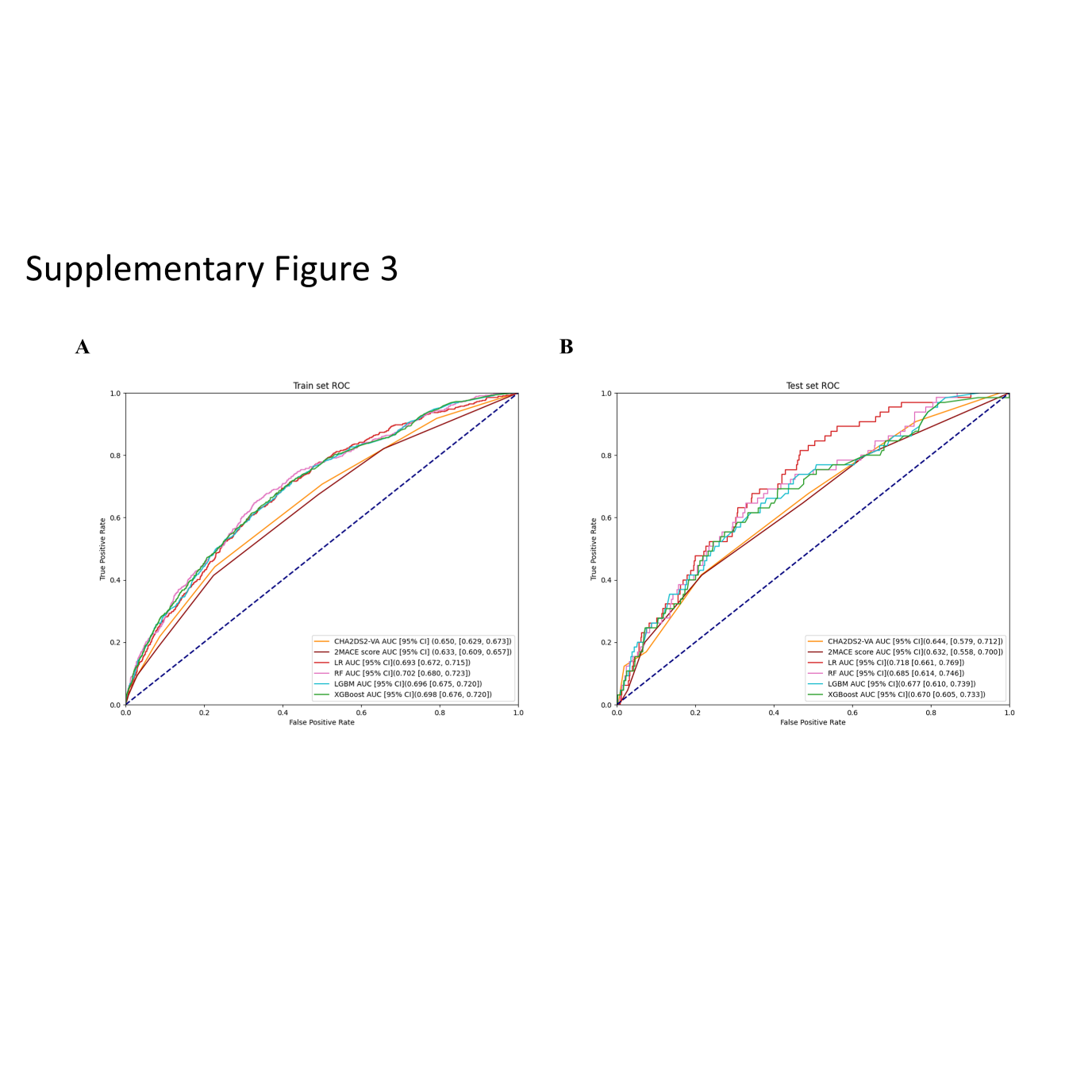


# Supplementary figure 3: Sensitivity analysis: ROC curves of prediction models in train set (A) and test set (B) in patients with over one year follow-up.

ROC, Receiver operating characteristic; AUC, areas under the ROC curve; CI: confidence interval; LR, Logistic Regression; RF, Random Forest; LGBM, Light Gradient Boosting Machine; XGBM, Extreme Gradient Boosting Machine

# Supplementary table 1: Baseline characteristic in train and test set

| Characteristic | Overall N = 15,8291 | Test set N = 1,5481 | Train set N = 14,281 | *P value* |
| --- | --- | --- | --- | --- |
| Age, years | 71 (65, 78) | 71 (65, 77) | 71 (65, 78) | 0.5 |
| eGFR, mL/min/1.73 m² | 76 (63, 90) | 75 (62, 90) | 76 (63, 90) | 0.5 |
| Male, n (%) | 8,642 (55%) | 863 (56%) | 7,779 (54%) | 0.3 |
| Diabetes, n (%) | 3,724 (24%) | 357 (23%) | 3,367 (24%) | 0.7 |
| Coronary artery disease, n (%) | 2,830 (18%) | 279 (18%) | 2,551 (18%) | 0.9 |
| Stable angina, n (%) | 959 (6.1%) | 87 (5.6%) | 872 (6.1%) | 0.4 |
| History of stroke, n (%) | 1,598 (10%) | 155 (10%) | 1,443 (10%) | >0.9 |
| Digoxin, n (%) | 1,387 (8.8%) | 151 (9.8%) | 1,236 (8.7%) | 0.15 |
| History of TE and MI, n (%) | 3,256 (21%) | 305 (20%) | 2,951 (21%) | 0.4 |
| Region North America, n (%) | 4,164 (26%) | 402 (26%) | 3,762 (26%) | 0.8 |
| OAC discontinuation, n (%) | 2,436 (15%) | 260 (17%) | 2,176 (15%) | 0.11 |
| Permanent AF, n (%) | 1,659 (10%) | 156 (10%) | 1,503 (11%) | 0.6 |
| Alcohol, n (%) | 4,574 (29%) | 442 (29%) | 4,132 (29%) | 0.8 |

Continuous variables were present by Median (IQR); Catalogic variables were present by frequency and percentage (n%).

eGFR: estimated Glomerular Filtration Rate; OAC, oral anticoagulation; IQR, interquartile range; TE, thromboembolism; MI, myocardial infarction;

# Supplementary table 2: Sensitivity analysis: Metrics to estimate models’ performance in patients with over one year follow-up.

|  | **LR** | **RF** | **LGBM** | **XGBM** | **2MACE score** | **CHA_2_DS_2_‑VA score** |
| --- | --- | --- | --- | --- | --- | --- |
| **Train set** |  |  |  |  |  |  |
| G mean | 0.213 | 0.218 | 0.213 | 0.213 | 0.188 | 0.195 |
| F1 Score | 0.120 | 0.132 | 0.117 | 0.118 | 0.097 | 0.100 |
| Accuracy | 0.606 | 0.672 | 0.583 | 0.590 | 0.516 | 0.507 |
| Precision | 0.066 | 0.074 | 0.064 | 0.064 | 0.053 | 0.054 |
| Recall | 0.692 | 0.646 | 0.711 | 0.705 | 0.674 | 0.707 |
| Specificity | 0.602 | 0.673 | 0.578 | 0.585 | 0.509 | 0.499 |
| AUC  [95% CI] | 0.693  [0.670, 0.715] | 0.702  [0.680, 0.723] | 0.696  [0.675, 0.720] | 0.698  [0.676, 0.720] | 0.633  [0.609, 0.657] | 0.650  [0.626, 0.673] |
| *P value*^1^ | < 0.001 | < 0.001 | < 0.001 | < 0.001 | 0.027 | *Reference* |
| **Test set** |  |  |  |  |  |  |
| G mean | 0.240 | 0.225 | 0.216 | 0.217 | 0.194 | 0.200 |
| F1 Score | 0.132 | 0.142 | 0.129 | 0.126 | 0.107 | 0.109 |
| Accuracy | 0.544 | 0.669 | 0.623 | 0.594 | 0.532 | 0.519 |
| Precision | 0.072 | 0.080 | 0.072 | 0.070 | 0.058 | 0.059 |
| Recall | 0.800 | 0.631 | 0.646 | 0.677 | 0.646 | 0.677 |
| Specificity | 0.533 | 0.671 | 0.622 | 0.591 | 0.526 | 0.512 |
| AUC  [95% CI] | 0.718  [0.661, 0.769] | 0.685  [0.614, 0.746] | 0.677  [0.610, 0.739] | 0.670  [0.605, 0.733] | 0.632  [0.558, 0.700] | 0.644  [0.579, 0.712] |
| *P value*^1^ | < 0.001 | 0.120 | 0.246 | 0.381 | 0.580 | *Reference* |

^1^ Delong tests were used to compare AUC

AUC, areas under the receiver operating characteristic curve; CI: confidence interval; LR, Logistic Regression; RF, Random Forest; LGBM, Light Gradient Boosting Machine; XGBM, Extreme Gradient Boosting Machine
